# Comparison of cancer incidence in Australian farm residents 45 years and over, compared to rural non-farm and urban residents - a data linkage study

**DOI:** 10.1186/s12885-017-3912-2

**Published:** 2018-01-05

**Authors:** Julie Depczynski, Timothy Dobbins, Bruce Armstrong, Tony Lower

**Affiliations:** 1 Australian Centre for Agricultural Health and Safety, The University of Sydney, Moree, Australia; 2 National Drug and Alcohol Research Centre, University of New South Wales, Sydney, Australia; 3 School of Global and Population Health, The University of Western Australia, Perth, Australia; 4 School of Public Health, The University of Sydney, Sydney, Australia

**Keywords:** Farm, Incidence, Cancer, Prostate, Breast, Melanoma, Lung, Colorectal, non-Hodgkin Lymphoma

## Abstract

**Background:**

It is not known if the incidence of common cancers in Australian farm residents is different to rural non-farm or urban residents.

**Methods:**

Data from farm, rural non-farm and urban participants of the 45 and Up Study cohort in New South Wales, Australia, were linked with state cancer registry data for the years 2006–2009. Directly standardised rate ratios for cancer incidence were compared for all-cancer, prostate, breast, colorectal cancer, melanoma and non-Hodgkin Lymphoma (NHL). Proportional hazards regression was used to generate incidence hazard ratios for each cancer type adjusted for relevant confounders.

**Results:**

Farm women had a significantly lower all-cancer hazard ratio than rural non-farm women (1.14, 1.01–1.29). However, the lower all-cancer risk observed in farm men, was not significant when compared to rural non-farm and urban counterparts. The all-cancer adjusted hazard ratio for combined rural non-farm and urban groups compared to farm referents, was significant for men (1.08,1.01–1.17) and women (1.13, 1.04–1.23). Confidence intervals did not exclude unity for differences in risk for prostate, breast, colorectal or lung cancers, NHL or melanoma. Whilst non-significant, farm residents had considerably lower risk of lung cancer than other residents after controlling for smoking and other factors.

**Conclusions:**

All-cancer risk was significantly lower in farm residents compared to combined rural non-farm and urban groups. Farm women had a significantly lower all-cancer adjusted hazard ratio than rural non-farm women. These differences appeared to be mainly due to lower lung cancer incidence in farm residents.

## Background

Registration of all cancers, excluding non-melanoma skin cancers, is a legal requirement in all Australian States [1, 2]. The most commonly diagnosed cancers in Australia include prostate, colorectal, breast, lung, melanoma and lymphoma [3]. The distribution of these cancers varies across rural and urban areas. Between 2005 and 2009, incidence of prostate, colorectal, breast cancer, melanoma and non-Hodgkin lymphoma (NHL) was highest in inner regional areas and lung cancer highest in very remote areas [3]. It has been suggested this reflects demographic variations, including age and socio-economic status; levels of engagement in risky behaviours such as smoking; and the availability or use of preventative health services in regional areas [4]. Cancer incidence is regularly reported by remoteness or accessibility to services [5], but not in a way that would distinguish those who do and do not live on farms. There is some limited information on mortality in male Australian farmers by occupation [6], however no information on cancer incidence for those who live on farms compared to others in rural areas or in cities in Australia, is known.

International studies have reported mixed findings on comparative cancer incidence between farmer and non-farmer groups. Most recent studies have reported reduced cancer incidence in farmers for all-cancer lung, breast and colorectal cancers [7–13], Possible reasons suggested include a healthy worker effect; greater levels of physical activity; differences in smoking rates; and the protective effects of farm endotoxin exposure [14]. Many studies of prostate and lympho-haematopoietic cancers have reported neutral findings. However, around one fifth of lymphoma studies, a quarter of prostate cancer studies and almost half of myeloma and leukaemia studies report significant excesses of cancer incidence in farmer groups [7–9, 11, 12, 15–26]. Pesticides and certain animal exposures are amongst the reasons suggested for the elevated risk of prostate and lymphohaematopoetic cancers [14, 27], However, findings also vary by location, study design and degree of control for confounders which can affect comparability and the strength of conclusions drawn [27, 28].

The current study aims to examine whether associations between cancer incidence and being a farmer or farm resident noted in other studies, are apparent in a large Australian cohort. From a rural health perspective, it also aims to differentiate the incidence of common cancers between farm residents and other rural people, not often specified in other studies. Findings may assist rural health programs better target cancer prevention intiatives; and/or highlight risk factors and exposures that require further investigation.

## Methods

This data linkage study was based on the 45 and Up Study cohort, consisting of 267,119 residents of New South Wales (NSW), Australia, aged 45 years and over. The cohort database is maintained and managed by the Sax Institute, in collaboration with health agency partners.^1^ This study assessed measures of cancer incidence for all-cancer, prostate, breast, colorectal, lung, melanoma and NHL, amongst farm, non-farm rural and urban residents, controlling for selected risk factors previously associated with cancer.

### Sampling and recruitment

Between January 2006 and December 2009, eligible NSW individuals 45 years and over, were randomly sampled from the Australian Department of Human Services database which provides near complete coverage of the NSW population. Persons aged 80 years and over and residents of rural and remote areas were oversampled by a ratio of 2:1. A pilot study was undertaken to validate recruitment procedures and refine survey questions. Subjects were mailed a questionnaire with a consent form for follow-up and data linkage to routine health databases. An additional 0.5% of the final cohort comprised volunteers who contacted the Study hotline to participate. The overall response rate for sampled individuals was 17–18%; representing approximately 11% of the NSW population aged 45 years and over [29]. The baseline questionnaire and further information about the study cohort is available from the *45 and Up Study* website [30].

### Datasets and linkage

Participant records from the 45 and Up Study cohort provided information on residence (farm, rural or urban), age, family history of cancer, household income, screening practices, diet, obesity, sun exposure, smoking and alcohol consumption. Cancer incident cases amongst participants were identified through linkage to the NSW Cancer Registry data, which contains records of all cases of cancer diagnosed in NSW residents, excluding non-melanoma skin cancers. Records were available for all new cancer notifications for the period 1st February 2006 (2006 was the first year in which 45 and Up Study participants were recruited) to 31st December 2009. Cancer type is derived and coded according to International Statistical Classification of Diseases Ninth Edition (ICD-9) cancer groupings [31]. Data quality control measures conducted by the NSW Cancer Registry are reported elsewhere [2]. A small proportion of cancer cases were identified and coded only on receipt of the Cause of Death Unit Record File.^2^ For the year 2009 only, delays in receiving deaths data is likely to have caused under-reporting of <1% to 3.2% of cancer incident cases for the cancers of interest [32].

**Fig. 1:**
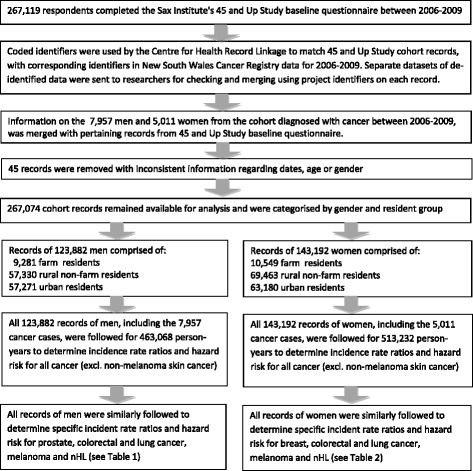
Summary of data linkage and flow of records by gender and resident group

Data linkage was conducted by the NSW Centre for Health Record Linkage, using a probabilistic record linkage method. Detailed information on data linkage methods is available elsewhere [33, 34]. Figure 1 provides a summary of data linkage and flow of participant records used to determine cancer incidence and risk. On reception of data, checks were undertaken for plausibility of dates and ranges, duplicate records, missing data, large numbers and illogical combinations of demographic, clinical variables and other unlikely combinations across datasets. Records with missing or invalid values for important variables of interest were excluded from analyses and noted in results (Table 1), where these represented more than 10% of the available sample. Records with inconsistent data resulting from transcription errors or false positive linkages which could not be resolved by cross-checking datasets, were also removed from analyses. Description of all survey questions, response coding and sample checking procedures can be found in relevant data dictionaries [31, 35].

**Table 1:** Cancer incidence and hazard ratios of farm, rural non-farm and urban men, 2006–2009

Cancer Type (ICD9)^a^	Cohort residence	Cases	Standardised	Adjusted	Hazard ratios (95% CI) for variables remaining in final model (excl. Age)/other notes
No. incident cases and total person-years	*n* = 123,882		(95% Cl)	(95% Cl)	
All malignant neoplasms (excl. non-melanoma skin cancer )	Farm *n* = 9281	496	91.7 (83.1–100.3)	1.00 (ref)	• Age^b^
C00 - C98 (excl. C44)	Rural non-farm *n* = 57,330	3814	102.9 (99.6–106.1)	1.09 (0.99–1.21)	• Smoking status
*n* = 7957 person-years = 463,068	Urban *n* = 57,271	3647	98.0 (94.8–101.2)	1.08 (0.97–1.20)	never HR = 1.00 ref.
					past HR = 1.09 (1.04–1.15)
					current HR = 1.11 (0.99–1.23)
					• Annual household income $70,000 and over ^c^
					yes vs no HR = 0.92 (0.86–0.98)
Prostate	Farm	252	98.5 (85.7–111.3)	1.00 (ref)	• Age ^b^
(C61)	Rural non-farm	1780	102.8 (98.0–107.6)	1.06 (0.93–1.21)	• Family history prostate cancer
*n* = 3647 Person-years = 470,298	Urban	1615	97.2 (92.4–102.0)	1.01 (0.88–1.16)	yes vs no HR = 1.83 (1.68–1.99)
					• Smoking status
					never HR = 1.00 ref.
					past HR = 0.96 (0.89–1.02)
					current HR = 0.80 (0.68–0.93)
Colorectal	Farm	62	100.3 (72.2–128.5)	1.00 (ref)	• Age^b^
(C18 - C21)	Rural non-farm	512	106.0 (96.7–115.3)	1.12 (0.86–1.46)	• Family history colorectal cancer
*n* = 1040 Person-years = 475,307	Urban	466	95.0 (86.3–103.8)	1.02 (0.78–1.33)	yes vs no HR = 1.31 (1.12–1.54)
					• Smoking status
					never HR = 1.00 ref.
					past HR = 1.30 (1.14–1.48)
					current HR = 1.26 (0.96–1.66)
Lung	Farm	19	71.4 (39.7–116.1)	1.00 (ref)	• Age^b^
(C34)	Rural non-farm	207	105.1 (90.6–119.5)	1.19 (0.70–2.02)	• Family history lung cancer
*n* = 426 Person-years = 476,776	Urban	200	95.6 (82.2–109.1)	1.22 (0.71–2.09)	yes vs no HR = 1.46 (1.06–2.01)
					• Smoking status
					never HR = 1.00 ref.
					past HR = 6.52 (4.31–9.87)
					current HR = 18.77 (11.72–30.05)
					• Annual household income over $70,000 ^c^
					Yes vs no HR = 0.48 (0.32–0.73)
Melanoma	Farm	75	108.5 (81.3–135.6)	1.00 (ref)	• Age^b^
(C43)	Rural non-farm	491	101.6 (92.5–110.6)	0.90 (0.71–1.15)	• Family history melanoma
*n* = 1051 Person-years = 475,264	Urban	485	98.4 (89.5–107.2)	0.87 (0.68–1.11)	yes vs no HR = 2.06 (1.71–2.47)
					• Smoking status
					never HR = 1.00 ref. past HR = 0.83 (0.73–0.94)
					current HR = 0.56 (0.40–0.79)
					• Tannability (response of skin repeatedly exposed to sunlight in summer without protection)
					very tan HR = 1.00 ref
					moderate tan HR = 1.52 (1.29–1.80)
					mild tan HR = 2.16 (1.80–2.59)
					never tan/freckle HR = 2.39 (1.90–3.01)
Non-Hodgkin Lymphoma	Farm	14	64.6 (34.8–109.2)	1.00 (ref)	• Age ^b^
(C82 - C85)	Rural non-farm	117	93.7 (76.6–110.8)	0.88 (0.50–1.55)	• Annual household income $70,000/year and over and red meat consumption remained as non-significant confounders
*n* = 269 Person-years = 476,768	Urban	138	106.5 (88.5–124.4)	0.96 (0.56–1.73)	

^a^ICD9 - International Statistical Classification of Diseases, Ninth Revision

^b^All models included age stratified by five-year age-bands at risk. Age remained significant in all models, but stratified hazard ratios are not reported

^c^Records with missing data on income constituted 16% of the available sample and were excluded from this model

### Definition of variables

‘Farm residents’ were defined as those who indicated that they lived in a ‘house on farm’ in the 45 and Up Study baseline questionnaire. Rural ‘non-farm’ and urban residents were further defined using the Accessibility/Remoteness Index of Australia (ARIA+) from postcode of residence at recruitment [5, 36]. Those participants whose ARIA+ classification indicated they did not live in a ‘Major city’, excluding those who also specified that they lived in a ‘house on farm’, were defined as rural non-farm residents. The remaining ‘urban’ participants were those who lived in a ‘Major city’. In population terms, this represents one of four cities in NSW with over 250,000 inhabitants. Where ARIA+ was not recorded, allocation was determined by cross-checking with postcode of residence and distances to treating hospitals in a linked hospital dataset.

Characteristics of the cohort in relation to risk factors of interest included age and others categorised in accordance with current national health or workplace recommendations [37–43]. These categories were: smoking status current, past, never; risky alcohol consumption >5 days/week, >2 drink/day; overweight and obesity status where Body Mass Index > 25; red meat consumption <3–4 < + serves/week; and weekday sun exposure <1–4 < + hrs/week). Tannability, the response of participants skin when repeatedly exposed to sunlight in summer without protection (never, mild, moderate, very), was considered for melanoma models only. Household income was stratified to approximate levels above or below the 2006 average annual household income of $70,000 in NSW in 2006 [44]. As a large survey collecting a range of health-related information from participants, family history information was only sought for some cancers. This included prostate, breast, colorectal, lung cancer and melanoma - but not NHL.

### Analytical procedures

Analyses were conducted using SAS 9.3™ [45]. software and Microsoft Excel 2007™ [46]. For incidence ratios, person-years were calculated commencing from 1st February 2006 to censorship date, or the 14th day of the month of notification of a selected cancer. Cancer-specific subsets were used for each cancer type, to allow for persons registered with more than one type of cancer. Person-years for each participant were split across 5-year age-bands at risk, commencing from age at recruitment, to enable allocation of risk time to each age strata, as the person aged [47, 48].

Consistent with *45 and Up Study* Collaboration recommendations, only internal comparisons between sub-groups within the cohort were made [29]. Direct age-standardisation methods were used to calculate standardised rate ratios for cancer incidence, appropriate for internal comparisons and where the age-structure of the sub-group is known [49]. The reference population used for standardisation was the whole *45 and Up Study.* Age-specific rates from each sub-group were used to derive an expected rate in the reference or standard population. This expected rate was divided by the standard population incidence rate and multiplied by 100 to derive a standardised rate ratio. Variance of observed counts were based on the Normal approximation or the Poisson distribution where less than 30 events occurred within a resident group [49]. Directly standardised rate ratios for cancer incidence were generated for all-cancers and the cancers of interest, being prostate, breast, colorectal, lung, melanoma and NHL.

Cox proportional hazards regression was used to model potential differences in incidence of selected cancers by cohort, controlling for risk factors [50]. Variables with univariate logrank *p* values < .25 were included in the base model. Interaction terms between variables in the base model and cohort were created and tested for effect modification. Models were progressively tested using backward elimination methods to the .05 level of significance; with non-significant variables also checked for confounding effects on the hazard ratios of cohort groups. Residual plots were examined to ensure assumptions of proportional hazards regression were met.

Sensitivity analyses were conducted using a smaller subset of data, where participants with prior cancer were removed and time to event for all records was limited to time since completing the questionnaire. However, results are primarily presented for the larger dataset, with reference to the comparison dataset as appropriate.

## Results

Information from the 45 and Up Study survey questionnaire was available for 267,119 participants. Forty five records were removed for which dates of recruitment, age or gender information across datasets was incomplete or inconsistent. The remaining 267,074 participants were followed for 1,006,229 person-years (mean 3.8 years /person, max 3.9 years). Standardised rate ratios for cancer incidence and adjusted hazard ratios by gender for each cancer are shown in Tables 1 and 2. Farm men in the cohort had an average age of *61.2 years (95%CI 61.0–61.4)*, compared to 63.7 years for rural non-farm (*95%CI 63.6–63.8)* and 64.3 years for urban counterparts (*95%CI 64.2–64.4)*. Similarly, the average age of farm women was 58.6 years (95%CI 58.5–58.8); younger than rural non-farm at 61.8 yrs. (*95%CI* 61.7–61.8) and 62.5 years for urban women (*95%CI* 62.4–62.5). Other summary characteristics of the cohort are available elsewhere [51].

**Table 2:** Cancer incidence and hazard ratios of farm, rural non-farm and urban women, 2006–2009

Cancer Type (ICD9)^a^	Cohort Residence	Cases	Standardised	Adjusted	Hazard ratios (95% CI) for variables remaining in final model (excl. Age) / other notes
No. incident cases and total person-years	*n* = 143,192		Rate Ratio (95% Cl)	Hazard Ratio (95% Cl)	
All malignant neoplasms (excl. non melanoma skin cancer)^b^	Farm *n* = 10,549	295	88.0 (75.8–100.2)	1.00 (ref)	• Age ^b^
C00 - C98 (excl. C44)	Rural non-farm *n* = 69,463	2472	102.0 (98.0–106.0)	1.14 (1.01–1.29)	• Overweight & obesity status
*n* = 5011 Person-years = 513,232	Urban *n* = 63,180	2244	100.1 (95.9–104.2)	1.12 (0.99–1.27)	yes vs no HR = 1.06 (1.00–1.13)
					• Smoking status
					never HR = 1.00 ref.
					past HR = 1.19 (1.12–1.27)
					current HR = 1.11 (0.98–1.25)
Breast	Farm	117	87.0 (69.6–104.5)	1.00 (ref)	• Age ^b^
(C50)	Rural non-farm	863	100.5 (93.8–107.3)	1.06 (0.87–1.29)	• Family history breast cancer
*n* = 1762 Person-years = 548,760	Urban	782	101.6 (94.5–108.8)	1.06 (0.86–1.29)	yes vs no HR = 1.58 (1.39–1.79)
					• Overweight & obesity status
					yes vs no HR = 1.13 (1.03–1.25)
					• Smoking status
					never HR = 1.00 ref.
					past HR = 1.11 (1.00–1.23)
					current HR = 0.78 (0.62–0.98)
					• Weekday sun exposure
					< 1 h HR = 1.33 (1.10–1.61)
					1–4 h HR = 1.12 (0.99–1.27)
					> 4 h HR = 1.00 ref.
Colorectal	Farm	48	97.3 (33.4–128.3)	1.00 (ref)	• Age^b^
(C18 - C21)	Rural non-farm	411	104.7 (94.9–114.9)	1.03 (0.76–1.40)	• Family history colorectal cancer
*n* = 815 Person-years = 550,743	Urban	356	95.4 (85.4–105.5)	0.95 (0.70–1.29)	yes vs no HR = 1.27 (1.07–1.52)
					• Smoking status
					never HR = 1.00 ref.
					past HR = 1.21 (1.06–1.41)
					current HR = 0.93 (0.65–1.33)
Lung	Farm	7	48.1 (8.8–117.8)	1.00 (ref)	• Age^b^
(C34)	Rural non-farm	157	106.3 (89.5–123.0)	1.82 (0.80–4.15)	• Smoking status
*n* = 300 Person-years = 551,870	Urban	136	101.8 (84.5–119.1)	1.83 (0.80–4.22)	never HR = 1.00 ref.
					past HR = 3.58 (2.54–5.07)
					current HR = 9.51 (6.17–14.65)
					• Annual household income $70,000 and over/yr remained as a non-significant confounder (*p* = .09)
Melanoma	Farm	41	103.3 (65.4–141.2)	1.00 (ref)	• Age^b^ *p* < .01
(C43)	Rural non-farm	332	110.3 (98.3–122.2)	1.11 (0.79–1.56)	• Family history melanoma
*n* = 622 Person-years = 551,022	Urban	249	89.6 (78.4–100.8)	0.95 (0.67–1.34)	yes vs. no HR = 2.08 (1.68–2.57) • Weekday sun exposure
					< 1 h HR = 1.20 (0.89–1.61)
					1–4 h HR = 1.00 ref
					> 4 h HR = 1.32 (1.09–1.60)
					• Tannability (response of skin repeatedly exposed to sunlight in summer without protection)
					very tan HR = 1.00 ref
					moderate tan HR = 1.46 (1.13–1.89)
					mild tan HR = 1.76 (1.35–2.29)
					never tan/freckle HR = 2.32 (1.74–3.09)
Non-Hodgkin’s Lymphoma	Farm	13	122.4 (55.5–210.0)	1.00 (ref)	• Age^b^
(C82 - C85)	Rural non-farm	93	91.8 (73.0–110.6)	1.15 (0.55–2.39)	• Annual household income $70,000 and over/yr remained a non-significant confounder (*p* = .62)
*n* = 211 Person-years = 551,833	Urban	105	108.8 (87.8–130.0)	1.30 (0.62–2.70)	

^a^ICD9 - International Statistical Classification of Diseases, Ninth Revision

^b^All models included age stratified by five-year age-bands at risk. Age remained significant in all models, but stratified hazard ratios are not reported

### Cancer incidence

All-cancer incidence rate ratios and the adjusted hazard of cancer diagnosis in farm men was almost 10% lower than in rural non-farm and urban men, although not significant between subgroups. However, when rural non-farm and urban men are combined as a group, the hazard ratio was 1.08 (1.01–1.17) compared to farm men. Farm men also had the lowest lung cancer and highest melanoma incidence, but these were not significantly different to either the rural non-farm or urban groups. There was little difference between farm men and other groups for NHL, prostate or colorectal cancer.

All-cancer incidence was lowest in farm women with the rate ratio 12–14% lower than either of the other groups, although differences were not significant. When controlling for other factors, the all-cancer adjusted hazard ratio for farm women was also 12–14% lower than other groups; being significantly lower than rural non-farm, but not urban women.. Similar to men, the hazard ratio relative to a combined rural non-farm and urban group was 1.13 (1.04–1.23), which was also significantly higher than that of farm women. There were no significant differences in rate ratios or adjusted hazard ratios between residence groups for any of the individual cancers tested. However, whilst confidence intervals did not exclude unity, both the incidence and adjusted hazard of lung cancer in farm women were around half that of other women. Results for the sensitivity analyses, which captured approximately 38% of cases across the selected cancers, were generally consistent with findings for the main analyses for both men and women and are reported elsewhere [51].

### Potential risk factors

Farm residents in this cohort were younger than rural non-farm and urban residents, with age controlled for in all adjusted hazard models. Family history was associated with most of the selected cancers, although this information was not available for NHL. Smoking status was significantly associated with lung cancer, with the adjusted hazard ratio for current smoking 18 times that of never smokers in men; and 9 times that of never smokers amongst women. In contrast, current smoking was negatively associated with prostate cancer and melanoma in men; as was income for lung and all-cancer in men. Tannability was negatively associated with melanoma in both genders. For women, higher sun exposure appeared weakly protective against breast cancer, but increased risk for melanoma. Overweight and obesity were associated with greater likelihood of both all-cancer and breast cancer in women. Income and red meat consumption were non-significant confounders for some cancers; whilst alcohol consumption was not associated with any of the cancers of interest.

## Discussion

Rate ratios for cancer incidence and adjusted hazard ratios are both discussed, although it is recommended more weight be given to the latter, as they control for additional risk factors. All-cancer incidence and adjusted hazard of a cancer diagnosis was lower in farm men, but differences were not statistically significant when compared to rural non-farm or to urban men separately. Farm women had non-significantly lower all-cancer incidence; but the adjusted hazard of a cancer diagnosis in farm women was significantly lower than rural non-farm women, controlling for other factors. There were no significant differences in either the standardised rate ratio or adjusted hazard ratio between cohorts for any of the individual cancers tested; although the incidence and adjusted hazard of lung cancer in farm women was around half that of other women. In this study, smoking was the most prominent modifiable risk factor in adjusted hazard models, having a particularly strong association with lung cancer. However, men who were current smokers were half as likely to be diagnosed with melanoma; and women with higher weekday sun exposure were least likely to be diagnosed with breast cancer.

### Incidence

Consistent with the direction of the findings, most reviews and recent studies have reported reduced all-cancer incidence in farmers [9, 11–14, 52, 53]. Some have attributed decreased cancer risk in farmers to a ‘healthy worker’ effect; a phenomenon observed when comparing occupational groups with the general population, that by nature exclude those who are unable to work for health reasons [9, 11, 54, 55]. Most farm businesses in Australia are family operations with ongoing generational commitment resulting in older farmers continuing to work into and past normal retirement age [56]. However, this study compared groups on a residential basis, which may have ameliorated occupational bias to some extent.

Comparative measures of smoking, alcohol and income-related risk factors for resident groups in this cohort presented elsewhere were generally more favourable amongst urban residents [51]. However, greater physical activity was suggested amongst farm residents, by their higher weekday sun exposure [51]. This may have contributed toward lower all cancer incidence, as suggested elsewhere [53].

Despite the small number of farm resident cases in this study for men and women, the lower lung cancer incidence and risk in farm residents support data from other studies reporting on farmers [7–13]. Lower smoking rates in farmers have often been suggested as the relevant factor, but this was not the case in this cohort, considering that urban men had lower current smoking rates [51]; and lower cancer incidence in farmers remained even after controlling for smoking in the analyses. Exposure to farm animals and environmental endotoxins have also been reported as possible explanations for lower lung cancer incidence in farmers, which remains a possibility here, although exposure information was not available and therefore not able to be assessed [57–60]. It is also possible differences in other, unmeasured risk factors, such as hormonal therapies, social characteristics and ethnicity, acted as potential confounders.

There was little discernible difference between groups in our study for the other selected cancers. Most recent studies of colorectal cancer in farmers have reported reduced incidence or risk in farmers. These have predominantly been large occupational cohort studies with a minimum follow-up of ten years [9–13]. Four of these studies reported reduced risk of breast cancer in farm women, as did two other studies of similar design [7, 8]. The only recent reports of excess breast and colorectal cancer in farm groups, have been from smaller case-control studies [61, 62].

Findings for breast, melanoma and prostate cancer in farmers have been mixed, with several reporting no significant differences between farm and non-farm groups [9, 11–13, 63–66]. Neutral findings have been reported for the majority of comparative studies of lymphoma in farmers published from 2008 to 2013 [9, 11–13, 20, 22, 24, 67–73]. However, more recent case-control studies have reported an excess of lymphomas in farm groups [15, 17, 18, 61], similar to earlier reviews of case control studies [52, 53, 74].

One prominent meta-analysis highlighted the inconsistencies of results brought about by variations in study design, risk measures, farmer definitions and geographic location [52]. A positive bias can occur in studies that use proportionate measures of risk in populations where the overall number of cases is small; and in case-control studies with non-population based controls [52]. This could help explain why such studies more often report increased prostate cancer and NHL risk in farmers, compared to cohort studies, which more often report neutral or reduced risk [52]. This effect was confirmed in a more recent review of prostate cancer risk in farmers published in 2014 [28]. Since then, two more studies reflecting these issues have reported opposing results; [6, 25] and a new meta-analysis limited to case-control studies, not unexpectedly reported higher risk in farmers [15]. In contrast, negative bias can be an issue in large cohort or occupational studies if there is limited information about possible confounders.

### Risk factors

Other studies have suggested increased cancer incidence in rural areas may be attributed to higher smoking and alcohol use, lower access to or utilisation of health services; and employment or income disadvantage [4, 75]. A greater proportion of rural non-farm residents in this cohort were current smokers and had lower incomes [51]. However, as expected when controlling for these factors, there was no evidence of a difference in lung cancer risk between rural non-farm and urban men in the adjusted model. In addition, whilst findings were not significant, these risk factors did not explain the lower likelihood of lung cancer in farm residents compared to the other groups. Confirmation of this effect with a larger farm resident sample is warranted.

Nevertheless, findings support what is already known about the hazardous effect of smoking upon lung cancer and all-cancer. It also supports the current health promotion priorities of Australia’s health systems with a focus on prevention and reduction of tobacco use, especially amongst groups with a higher prevalence of smoking [76]. The negative associations between smoking and breast cancer, prostate cancer and melanoma in men may have been an artefact of the relatively short follow-up period. However, a recent meta-analyses has also reported negative links between smoking and prostate cancer incidence and unclear links to breast cancer [77, 78]. There have been reports of negative associations between smoking and melanoma - although the biological mechanisms are unclear [79–81]. Overall, the negative associations with smoking had a relatively minor impact upon the relative patterns of risk between resident groups.

A related study of cancer mortality risk in this cohort, found that compared to very low exposure, weekday sun exposure of 1–4 h was protective against NHL, prostate, breast, melanoma and lung cancer mortality [51]. This was also the case for melanoma incidence in this study. Others have similarly reported inverse melanoma risk with occupational or weekday patterns of sun exposure, as opposed to the more intermittent patterns giving rise to sunburn that raises melanoma risk [82]. However, 4 h + sun exposure was most protective against breast cancer. Other studies have also suggested links between sun exposure, Vitamin D levels and reduced risk of breast cancer [83–85]. However, it is also possible that moderate sun exposure represented greater relative health and outdoor physical activity, which is promoted in Australian cancer prevention guidelines [37].

Several studies have explored positive associations between cancer incidence and farm environmental exposures, such as pesticides. However, these are outside the scope of this study, as they do not generally compare farm and non-farm groups; and farm exposure information was not available in this dataset.

The negative significant association between lung cancer in men and income, is consistent with findings elsewhere, relating to higher levels of smoking in lower socio-economic groups [86]. Overweight and obesity was associated with breast cancer in this study, also consistent with reports in the health literature [37]. However, contrary to evidence of links between alcohol consumption and breast, colorectal and other cancers, this was not associated with any of the selected cancers in this cohort [37].

### Limitations

There are a number of limitations in this study that may have affected the results. Firstly, data on incident cases at the time the research was conducted were only available for a relatively short period of follow-up, resulting in low power and wide confidence intervals for some analyses. This may have impacted upon the significance of some findings, favouring a bias toward the null. Discussion of results with confidence intervals that include unity should be considered exploratory; and larger, consistent differences given more weight. Nevertheless, results still offer insight into potential differences and guidance for further work.

In addition, to maximize both cases numbers and follow-up time, this study included all records of cancer for participants who could potentially receive a diagnosis of cancer at any time in the 2006–2009 study period; that is, cancer diagnosis in some participants could have preceded their enrolment in the 45 and Up Study. However, such an effect is likely to be non-differential relating to residence; and results of the sensitivity analyses were consistent with and support the main findings.

The need to exclude records with missing variable information from models may have impacted upon the results, although this is not likely to have been differential across groups or between cases and non-cases. Other limitations include the potential mobility of participants regarding their residential status and that only the more commonly known risk factors were considered for analyses. A myriad of other potential risk factors and confounders were not measured (e.g. social factors, ethnicity); and may have contributed to the differences observed.

The 45 and Up Study, even with its robust sampling methods, is not necessarily representative of the population of NSW aged 45 and over [29]. However, it is one of the largest cohorts of its kind in the world; and there was little evidence of selection bias observed when associations between risk factors and disease in the 45 and Up Study population, were compared with those of another population-based dataset drawn from the same population using different methods [87]. Over-sampling in rural areas to ensure representation of smaller population groups, is also likely to have minimised selection-bias at sub-group level. However, only internal comparisons between sub-groups have been made in this study, previously documented as valid and the most appropriate [29]. Caution is therefore advised with the generalisation of results.

The definition of a ‘farm resident’ in this study was also open to respondents’ interpretation of ‘farm’, which could include small holdings used for commercial, recreational or both purposes. Exposures could be quite different depending on which of these purposes was dominant. Farm exposure differences and errors arising from misclassification of residence, are likely to have lessened any differences between resident groups, but not likely to have systematically affected non-residential risk factors. Therefore, any potential bias is likely toward the null and an underestimation of a relationship between farm residence and cancer incidence.

## Conclusions

This study is the first to examine differences in incidence of cancer between farm, rural non-farm and urban residents in Australia. Controlling for a range of risk factors, farm women had a significantly lower hazard ratio for cancer diagnosis than rural non-farm women. Farm men also had lower risk of cancer diagnosis, but this was not statistically significant compared to rural non-farm and urban men. When combining rural non-farm and urban groups, the all-cancer adjusted hazard ratio was significantly lower in both farm men and women, due to increased precision.

Differences between groups in the risk of prostate, breast, colorectal or lung cancers, NHL and melanoma were not significant after controlling for commonly known risk factors. However, notwithstanding small case numbers and a lack of statistical significance, farm women had around half the risk of other women in being diagnosed with lung cancer; controlling for smoking and other factors. Differences in all cancer risk appeared to be mainly due to lower lung cancer incidence in farm residents.

## Abbreviations

ARIA +: Accessibility/Remoteness Index of Australia
ICD-9: International Statistical Classification of Diseases Ninth Edition
NHL: non-Hodgkin Lymphoma
NSW: New South Wales

## Footnotes

1 The *45 and Up Study* is managed by the Sax Institute in collaboration with major partner Cancer Council NSW; and partners: the National Heart Foundation of Australia (NSW Division); NSW Ministry of Health; NSW Government Family & Community Services – Ageing, Carers and the Disability Council NSW; and the Australian Red Cross Blood Service; and thanks to the many thousands of people participating in the 45 and Up Study.

2 The Cause of Death Unit Record File (COD URF) is held by the NSW Ministry of Health Secure Analytics for Population Health Research and Intelligence and provided by the Australian Coordinating Registry for COD URF on behalf of Australian Registries of Births, Deaths and Marriages, Australian Coroners and the National Coronial Information System.
